# Up-gradation of the dielectric, physical & chemical properties of cottonseed-based, non-edible green nanofluids as sustainable alternatives for high-voltage equipment's insulation fluids

**DOI:** 10.1016/j.heliyon.2024.e28352

**Published:** 2024-03-24

**Authors:** Abubakar Siddique, Muhammad Adnan, Waseem Aslam, Hafiz Ghulam Murtaza Qamar, Muhammad Nadeem Aslam, Salman A. Alqahtani

**Affiliations:** a Department of Electrical Engineering, Khwaja Fareed University of Engineering and Information Technology (KFUEIT), Rahim Yar Khan, 64200, Pakistan; b Department of Electrical Electronics and Computer Systems, University of Sargodha (UOS) Sargodha, Punjab, 40100, Pakistan; c Department of Electrical Engineering, Yanshan University China; d Department of Electrical Engineering, Institute of Southern Punjab (ISP), Punjab, Pakistan; e Department of Computer Engineering, College of Computer and Information Sciences, King Saud University P.O.Box 51178, Riyadh, 11543, Saudi Arabia

**Keywords:** Nanofluids, Natural easter oil, Transformer oil, Thermal conductivity, Di-electric properties, Cottonseed oil, Nanoparticles, Insulation oils, Biodegradable fluids, Stability

## Abstract

The use of natural ester oils as electrically insulating fluids has gained significant attention from industries and electrical utilities as they aim to replace traditional mineral oils. However, most natural ester oils are derived from edible products, which has the potential to contribute to the food crisis. Therefore, nonedible green nanofluids made from cottonseed oil (CSO) have been targeted as a keen solution to this issue. However, Al_2_O_3_, TiO_2_, Fe_2_O_3_, SiO_2_, and graphene nanoparticles at (0.025, 0.05, and 0.075 wt/vol%) were used as additives, along with surfactant Olic Ac-id and Ethanol (1:5) due to their promising impact on the dielectric and thermal properties of the nanofluid. The nanofluid synthesis process was practically conducted in HV & Chemical Laboratories using one-step and two-step methods, and their breakdown voltage results and chemical properties (e.g., fire point, flash point, cloud point, pour point, viscosity, acidity, moisture content, resistivity, and dissipation factor) were compared. The physical mechanisms underlying these properties were also analyzed and tested. For the validation of the proposed vegetable oil the results have been compared with traditional mineral oil for high-voltage equipment's. The findings suggest that the proposed nonedible green nanofluids-based cottonseed oil (CSO) has a high potential to be used as electrically insulating fluids, providing a sustainable alternative to conventional mineral oils. Overall, this study provides insights into the use of non-edible green nanofluids as a solution to the potential contribution of natural ester oils to the food crisis. The findings highlight the importance of sustainable solutions in the energy industry and the need for further research in this area.

## 1 Introduction

Oil-immersed high voltages typically use insulating fluids that are based on mineral oil (MO). Mineral oil (MO) has a dual functionality, serving both as an efficient dielectric material and a cost-effective cooling medium. Nonetheless, the sustainability of transformer oil has become a contentious issue, primarily due to the finite resources of mineral oil derived from petroleum products. Furthermore, mineral oil (MO) presents various notable drawbacks, such as limited biodegradability (<30%), low flash and fire points, a restricted moisture content threshold, and non-compliance with health and environmental regulations [1].

The challenges encountered have sparked a growing interest in innovative alternatives crafted from easily obtainable resources, showcasing substantial long-term potential. One of the most encouraging replacements involves the utilization of vegetable oil (VO) in the shape of natural esters as insulating fluids. Numerous researchers and industries are actively investigating the application of natural esters as insulating oils in high-voltage scenarios [2,3].

The obstacles linked with conventional mineral oils as insulation fluids have generated increased attention towards alternative solutions crafted from readily accessible resources, showcasing substantial long-term potential [1]. A particularly promising substitute involves the utilization of natural esters, sourced from vegetable oils (VO), as insulation fluids. Researchers and industries are exploring the use of natural esters, such as BIOTEMP®, Envirotemp®FR3TM, MIDEL 1204, MIDEL 1215, and fabricate esters such as MIDEL 7131, as insulating oils in high voltages [2,3].

These different esters vary in their properties, such as viscosity, density, thermal conductivity, and BDV, which are primarily due to the source of the ester, whether natural or synthetic and the constituents that comprise them. Most natural esters contain at least minimum of 95% base as VO, with other percentage made up of supplement for enhance properties, according to the IEEE Standard C57.147 [4]. Natural esters have demonstrated beneficial performance with respect to some practical issues, such as higher dielectric strength and higher tensile strength of papers impregnated with these esters under various aging conditions, compared to those impregnated with mineral oil (MO) [[5], [6], [7]]. MIDEL 1204 and MIDEL 1215 have also been found to show lower annual loss of life than MO, while MIDEL 7131 showed lowest annual loss of the life among all.

Although VO have the potential to serve as better transformer-insulating oils, their commercial use is hindered by certain challenges. The presence of unsaturated fatty acids one of these challenges, which can render oils unstable and prone to oxidation, thereby negatively affecting their properties. To address this issue, antioxidants have been added as additives to vegetable oils (VO) to improve their critical properties, particularly under conditions of thermal aging [8,9]. In addition, the extraction of vegetable oils (VO) from edible products can deplete the food supply, which poses a significant challenge to their widespread use as insulating fluids. The challenge of increasing oil recovery in flooded production wells, emphasizing the need to limit water inflow through advanced technologies. The sediment-gelling composition (SGC) technology is explored for efficient bottom-hole zone treatments, involving the creation of an insoluble sediment and polymer gel to block water-washed intervals. Despite SGCs' potential, their success in waterproofing works is not yet optimal, and the study suggests improving effectiveness by selecting SGCs more thoughtfully for specific well conditions. The efficiency of SGC treatments relies on good selective filterability, controllability of water-shedding, and consideration of rheological and filtration characteristics. Given the prevalent issue of increasing water cut in late-stage oil fields globally, controlling water cut becomes crucial, requiring comprehensive studies and diagnostic methods to address the complexity of excess water supply. The paper emphasizes the need for complex studies, including an analysis of geological conditions and rheological characteristics, to enhance the effectiveness of water inflow limitation technologies. The scientific novelty lies in experimental studies establishing the influence of polymer and alkali concentrations on the sedimentation mechanism, providing valuable insights for improving waterproofing works [10,11].

Pakistan is expected to become the fastest-growing market in the energy sector, with a focus on the expansion of power transmission and distribution (PTD) infrastructure. This growth has led to an increasing demand for high voltages, resulting in a rising market for transformer oil. At the same time, Pakistan is ranked as the world's third-largest cotton producer [[12], [13], [14]], providing an abundant supply of non-edible cottonseed oil (CSO) (CSO). As a result, there is a significant opportunity in Pakistan to utilize this non-edible CSO as a feedstock to produce commercial standard insulating fluid for use in transformers.

Cottonseed oil (CSO) is an environmentally friendly option as it is biodegradable and non-toxic [15]. Additionally, it has a high flash & fire point, which means it is less flammable than MO. All the same, due to its high proportion of unsaturated fatty acids, CSO is prone to oxidation. To mitigate this tendency towards oxidation, CSO can be upgraded to have a higher oleic content [16] or have antioxidants added. Despite its potential as an alternative insulating fluid in transformers, very few studies have explored the use of CSO. However, some studies have demonstrated its compatibility with transformer parts, and its acid and copper strip corrosion values meet standard specifications [17]. It should be noted that CSO has lower BDV compared to other natural esters [18].

In recent times, the furthermore addition of nanoparticles has been suggested as a means of modifying insulating fluids, resulting in the formation of nanofluids, to enhance their thermal and dielectric properties [[19], [20], [21], [22]]. The effectiveness of nanofluids depends on several factors such as the type, size, shape, and agglomeration rate of the particles as well as the filling percentage [[23], [24], [25], [26], [27]]. Nanofillers can be categorized into metals, metal oxides, and carbon-based materials. The use of metal oxides as nanofillers can enhance the electrical properties of base fluids [28,29]. On the other hand, the use of carbon-based nanofillers can enhance the thermal properties of the fluids. Among these nanofillers, carbon nanotubes were gaining prominence, even at low filler percentages [30]. CNTs have a one-dimensional structure with the van der Waals π-π stacking interfaces, which accelerates agglomeration between carbon nanofiller tubes fibrils. To prevent CNT aggregation must be functionalized with strong acids, which creates unintentional defects in the uninterrupted sp2-bonded carbon structures. These defects enhance phonon dispersion, which significantly reduces thermal conductivity [31,32].

To solve these issues, researchers have turned to the development of two-dimensional (2D) structures such as graphene [33,34], graphene oxide, a hexagonal carbon lattice with 2D properties. The sp2 covalent bonding of the carbon atoms in graphene creates an extremely high thermal conductivity [35]. Moreover, the 2D nature of carbon materials con-tributes with the superior electrical insulation [36] and making them desirable nanofillers for enhancing overall properties of high voltage oil.

This study seeks to address the limitations of natural esters derived from edible sources by exploring the potential of using cottonseed oil (CSO) as an alternative for high voltage insulation. The study proposes the incorporation of nanoparticles such as Al_2_O_3_, TiO_2_, Fe_2_O_3_, SiO_2_, and graphene (at a concentration of 0.025 wt/vol%), along with a surfactant mixture of Olic Acid and Ethanol (in a ratio of 1:5) to enhance, the dielectric and thermal properties of the oil. These additives have been selected based on their demonstrated ability to enhance the desired properties of the resulting nanofluid. The nanofluid synthesis process was conducted using one- and two-step methods, and their breakdown voltage results and chemical properties (e.g., fire point, flash point, cloud point, pour point, viscosity) were compared. A detailed description of the synthesis was presented. Different nanofillers were introduced at the same concentration with both one and two-step methods for comparison of their results. The study conducted extensive dispersion stability analyses of cottonseed nanofluids and evaluated their dielectric & thermal properties in comparison to base CSO. The obtained results were discussed to uncover the fundamental mechanism.

The crucial role of transition metal oxides, particularly ferrites, in various technological applications. Strontium hexaferrites stand out for their exceptional microwave properties, making them suitable for high-frequency applications. The combination of soft and hard magnetic phases, explored in nanosized particles, has garnered significant attention due to their potential in creating high-energy products for permanent magnets and radio materials. The study focuses on synthesizing and analyzing SrNi0.02Zr0.01Fe11.96O19/CoFe2O4 nanocomposites, aiming to understand the impact of increasing CoFe2O4 concentration on their structure, morphology, and magnetic and microwave properties [37]. The significance of complex magnetic oxides in contemporary science, particularly ferrites, with diverse structures like spinel's and hexaferrites, each exhibiting unique magnetic and electrical properties. The exchange coupling of soft and hard magnetic phases gives rise to exchange-spring magnets, demonstrating high coercivity and saturation magnetization. This interaction enhances properties for applications in biomedicine, spintronics, and nanoelectronics. The study explores the synthesis and structural aspects of CoFe2O4/Fe3O4 exchange-spring magnets, emphasizing their superior energy product and stability. Additionally, it investigates rare earth-substituted hard-soft ferrite NCs, shedding light on their electrical and dielectric properties. The current research aims to synthesize and analyze nanocomposites (CFOx/NCZOy) derived from nanosized spinel ferrites, offering valuable insights into their structural parameters and microwave properties under exchange coupling [38]. The surge in publications on multicomponent oxides underscores their scientific importance, with a focus on magnetic iron oxides, particularly ferrites. Spinel's and hexaferrites, known for soft magnetic behavior, exhibit applications in optics and high-frequency domains. The paragraph explores the distinct properties of exchange-coupled nanocomposites (NCs), emphasizing the synergistic effects in hard/soft (H/S) and soft/soft (S/S) NCs. Additionally, it touches on the eco-friendly green synthesis of nanomaterials, highlighting the use of natural extracts. The study introduces novel insights into the chemical and magnetic characteristics of “soft/soft” functional NCs [39].

Hexaferrites, known for their hexagonal crystal structure, are utilized as permanent magnets and microwave materials due to their exceptional magnetic and chemical stability. Recent findings suggest spontaneous electrical polarization in hexaferrites, classifying them as magnetically stimulated ferroelectrics. M-type hexaferrites demonstrate coexistence of magnetic and electric ordering, challenging conventional explanations. Investigations into BaFe12O19 single crystals reveal dynamic disorder near pentahedral positions, leading to a polar phase at lower temperatures. Quantum paraelectric properties in (Ba, Sr) Fe12O19 defy the d°-ness rule. Diamagnetic substitution, explored in this study with indium-doped BaFe12−xInxO19 hexaferrites, aims to engineer ferroelectric properties and elucidate magnetoelectric effects over a broad temperature range (4–730 K) [40,41]. the magnetic and dielectric performance of barium hexaferrites by substituting titanium cations at high concentrations (up to 3.00), clarifying the occupied positions of titanium cations. Crystal structure parameters were determined using the Rietveld method from X-ray powder diffraction data. Investigations included spontaneous magnetization, remnant magnetization, magneto crystalline anisotropy coefficient, anisotropy field at temperatures of 5 K and 300 K, and electrical ac-resistivity, real part of permittivity, and tangent loss across a wide temperature range and different frequencies. The study holds significance for enhancing microwave absorption properties [42]. the crystal structure, magnetic properties, and microwave characteristics of strontium hexaferrites substituted with In3+ cations. Strontium hexaferrites exhibit significant potential for microwave applications due to their complex dielectric and magnetic properties. Investigations include cationic substitution, co-substitution with titanium and zinc, and the formation of composites with graphene, all contributing to altered microwave properties. The charge state of paramagnetic iron cations plays a crucial role, influencing the electromagnetic behavior of the hexaferrite. Mössbauer studies confirm the filling of 4fVI octahedral positions by In3+ cations, impacting saturation magnetization and coercive force. The study extends to the attempt of describing the crystal structure within no centrosymmetric space groups to explain ferroelectric properties, highlighting the ongoing discussions on the reasons for spontaneous polarization in indium-substituted strontium hexaferrites. The research combines macro measurements, powder neutron diffraction, and waveguide methods to provide comprehensive insights [43].

## 2 Materials and methods

### 2.1 Materials

Cottonseed was bought from the farmer in Rahim Yar Khan, Pakistan then extracted the oil in Sar Sabz Oil Mills Ltd., Abu Dhabi Road, RYK. The nanoparticles namely silicon oxide (SiO_2_), titanium oxide (TiO_2_), ferric oxide (Fe_2_O_3_), aluminum oxide (Al_2_O_3_), and graphene were imported from China for synthesis. Table 1 shows the physical chemical properties of base oil while Table 2 shows the characteristics of nanoparticles. Some characteristics were not provided from supplier that we take from the literature.

**Table 1:** Physical chemical characteristics of cottonseed oil.

Properties	Cottonseed Oil (CSO)
Density at 20 ¬0C (kg/dm3)	925
Viscosity (cSt)	35
Pour point (0C)	−6
Flash point (0C)	215
Fire point (0C)	250
Acidity (mg KOH/g)	0.15
Moisture content (ppm)	0.26
Resistivity (GΩ.m)	20
Dissipation factor at 90 0C	0.03

**Table 2:** Properties of the nanoparticles.

Nanoparticles	SiO_2_	TiO_2_	Al_2_O_3_	Fe_2_O_3_	Graphene
Size (nm)	10	10	20	50	20
Specific Surface Area (m2/g)	272.7	153.8	75.4	5.6	–
Density (g/cm3)	2.2	3.9	3.98	5.24	1.9–2.2
Dielectric Constant	3.9	80	8–12	12	–

### 2.2 Preparation of base oil

The preparation of the base oil for the development of nanofluid-based vegetable oil (VO) for high voltages involves a multi-step process. Initially, seeds of cotton are procured, and oil is extracted using a milling process. The extracted oil is then subjected to three different preparation methods: unrefined, refined, and filtered.

The unrefined method involves using the oil without any filtration or refining. This sample was tested in the high voltage laboratory, and it was observed that its breakdown voltage was 23.66 kV. However, this value is relatively low for a high voltage application.

The refined method involves the industrial process of refining the oil to make it useable. This sample was also tested in the high-voltage laboratory, and the breakdown volt-age observed was only 5.10 kV. This low value was due to the breaking of oil molecules caused by the high temperature in the boiler during the refining process.

To overcome this issue, the oil was filtered using filter paper. The filtration process was carried out in the laboratory, and the oil sample was again tested in the high-voltage laboratory. The result of the test showed that the breakdown voltage was increased to 27.34 kV, making it suitable for high voltage application.

**Fig. 1:**
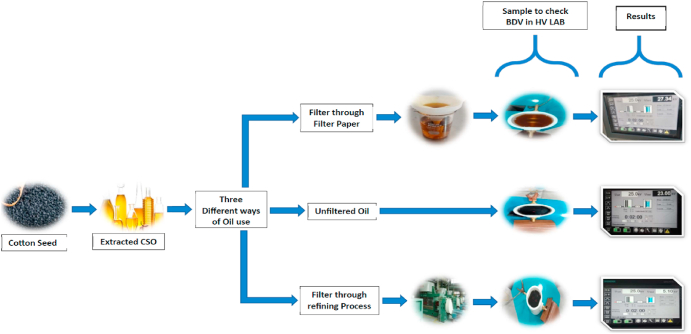
Preparation of Base Oil & Select the best way by Compere the possible method that was use.

The literature review and discussions with chemistry and chemical engineering experts helped me understand the underlying reasons for the difference in the breakdown voltage values. So, the filtered oil is better because it has high break down voltage strength as shown in Fig. 1.

### 2.3 Nanofluid preparation

Nanofluids are produced by adding nanoparticles to base oil using either one-step or two-step method. This study examined both methods for preparing nanofluids at concentrations of 0.025, 0.55, and 0.075 g/ml% and compared their electrical and chemical proper-ties.

**One-Step Method.** The one-step method is a straightforward and cost-effective method for preparing nanoparticles. It involves adding the nanoparticles directly to the base oil and stirring vigorously until a homogeneous mixture is obtained. This technique finds widespread use in the industry for the large-scale production of nanoparticles.

**Fig. 2:**
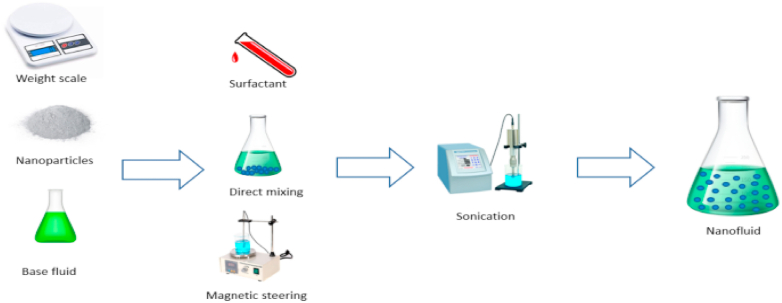
Experimental process of one step method.

The roots of the one-step method can be traced back to the 1960s, marking the initiation of research into nanoparticle synthesis using this approach. Over the years, the method has undergone various modifications aimed at enhancing both the synthesis process and the properties of the nanoparticles. A notable advantage of the one-step method lies in its relative simplicity compared to alternative nanoparticle synthesis techniques. It eliminates the need for intricate equipment or extensive purification steps, rendering it an appealing choice for large-scale nanoparticle production. However, the method does come with certain limitations, including the necessity for precise control of reaction conditions to prevent agglomeration and the challenge of achieving a uniform dispersion of nanoparticles in the base oil. Despite these constraints, the one-step method continues to be a favored and widely employed approach for nanoparticle preparation, especially in industrial applications. In our study, we employed the one-step method, as illustrated in Fig. 2, to synthesize five distinct nanoparticles: aluminum oxide, silicon oxide, titanium oxide, ferric oxide, and graphene oxide.

To create the nanofluids, we employed a straightforward mixing procedure. Initially, the base oil, cottonseed oil, was heated to 60 °C using a hot plate equipped with a magnetic stirrer. Once the desired temperature was reached, the nanoparticles Al_2_O_3_, TiO_2_, Fe_2_O_3_, SiO_2_, and graphene were accurately weighed and introduced into the oil. The mixture underwent stirring for 2 h at a consistent speed of 500 rpm to ensure the even dispersion of nanoparticles in the oil. Following the completion of stirring, the nanofluids were allowed to cool at room temperature and then stored in a sealed container for future use. The concentration of nanoparticles in the nanofluids was set at 0.025 wt/vol%. This uncomplicated mixing process facilitated the easy preparation of nanofluids without requiring specialized equipment or intricate procedures.

For the preparation of nanofluids at a 0.05 wt/vol% concentration, a similar simple mixing process was utilized. Initially, the nanoparticles (Al_2_O_3_, TiO_2_, Fe_2_O_3_, SiO_2_, and graphene) were dispersed in ethanol using a magnetic stirrer for 30 min. Subsequently, cottonseed oil was heated to 60 °C, and the nanoparticle-ethanol suspension was gradually added into the oil while stirring for 30 min using a mechanical stirrer. The resulting mixture underwent sonication for 60 min to guarantee the uniform distribution of nanoparticles in the oil. The sonication process employed an ultrasonic processor (50 W, 20 kHz) with a 10 mm probe. The resultant nanofluids were stored at room temperature for subsequent testing. The one-step simple mixing method demonstrated effectiveness in producing stable nanofluids with good dispersion, eliminating the need for surfactants or stabilizers.

For the 0.075 wt/vol% concentration, the same one-step method was applied to prepare nanofluids of cottonseed oil with Al_2_O_3_, TiO_2_, Fe_2_O_3_, SiO_2_, and graphene nanoparticles. The nanoparticles were weighed and dispersed into the base oil with the assistance of a magnetic stirrer for 1 h at room temperature. The resulting mixture underwent 30 min of sonication to achieve a homogeneous nanofluid. Characterization of nanoparticle size and stability in the nanofluids was conducted using DLS and zeta potential measurements. The nanofluids were then subjected to dissipation factor testing using an LCR meter within a frequency range of 1 kHz to 1 MHz. Results indicated that nanofluids with Al_2_O_3_, TiO_2_, and SiO_2_ nanoparticles exhibited a decrease in dissipation factor compared to the base oil, while those with Fe2O3 and graphene nanoparticles displayed an increase. The temperature range for the experiments was set between 25 °C and 60 °C. Overall, the simple mixing process in the one-step method proved effective in producing stable nanofluids with enhanced thermal properties at different concentrations.

**Two-Step Method.** The two-step method constitutes an alternate approach for nanoparticle preparation. In this process, the initial step involves synthesizing a precursor solution, serving as the foundation for the subsequent step where the actual formation of nanoparticles takes place. A notable advantage of the two-step method lies in its capacity to afford better control over the size and shape of the nanoparticles. This is attributed to the customization of the precursor solution under specific conditions conducive to the formation of particular nanoparticle types [44,45].

The two-step method encompasses various techniques, including the sol-gel method, microemulsion method, and hydrothermal method. The sol-gel method entails utilizing a sol (colloidal suspension), which is then gelatinized to yield a solid material. The microemulsion method involves employing a mixture of two immiscible liquids, forming nanodroplets in the presence of a surfactant. The hydrothermal method utilizes high-temperature and high-pressure conditions to facilitate nanoparticle growth.

**Fig. 3:**
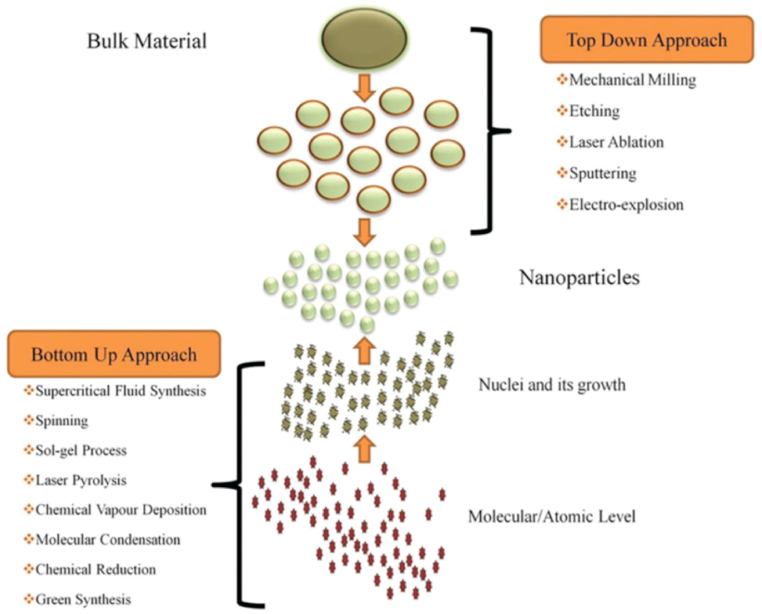
Synthesis of nanoparticles.

Each approach bears its unique advantages and drawbacks. For instance, the sol-gel method is relatively straightforward and yields highly pure nanoparticles, albeit posing challenges in controlling particle size and shape. The microemulsion method offers versatility and produces nanoparticles with well-defined sizes and shapes but necessitates specialized equipment. The hydrothermal method is effective in generating nanoparticles with high crystallinity and uniformity, albeit presenting difficulties in controlling particle size and shape. The synthesis of precursor solutions varied depending on the nanoparticle type, and subsequent nanoparticle formation employed diverse techniques such as calcination and hydrothermal treatment, as depicted in Fig. 3.

Characterization of the nanoparticles produced through the two-step method involved various techniques, including transmission electron microscopy, X-ray diffraction, and Fourier transform infrared spectroscopy. Results demonstrated the efficacy of the two-step method in producing nanoparticles with high crystallinity and uniformity. The breakdown voltage of nanofluids prepared with these nanoparticles was determined to be 38 kV, surpassing that of the base oil alone.

**Fig. 4:**
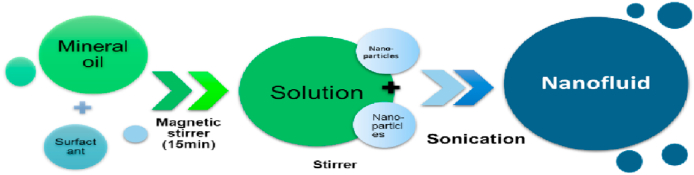
Experimental methods of two step method.

In this research, we used five different nanoparticles, namely aluminum oxide, silicon ox-ide, titanium oxide, ferric oxide, and graphene oxide, and adopt the sol-gel method, in two-step method according to Fig. 4.

The preparation procedure utilized the two-step method, wherein the nanoparticle syn-thesis and NFs preparation were isolated. For each NF, the following steps were strictly followed: (a) micro membrane filter purification of the base liquid, (b) adding 0.75 wt% of the oleic acid and mixing it with a high shear mixer (HS) at 13,000 rpm for 5 min, (c) adding the desired amount of powder nanoparticles to the base oil and agitating the resulting mixture for the 20 min using HS mixer, (d) Lastly, subjecting nanofluids samples in ultrasonication for the 120 min to enhance NP dispersion. A total of five NFs, each with a volume of 300 mL and NPs concentrations of 0.025, 0.05 and 0.075 g/mL, were prepared. The ultra-sonication device utilized for the experiment had a power of 500 W, operating at a frequency of 20 kHz, and was equipped with the 25 mm solid probe. It operated with a 67% duty cycle in pulsed mode and 15 s of the period, set at 60% amplitude. The device was rested for 10 min after every 30 min of use to prevent overheating & damage to the solid probe.

### 2.4 AC breakdown voltage of cottonseed oil

**Fig. 5:**
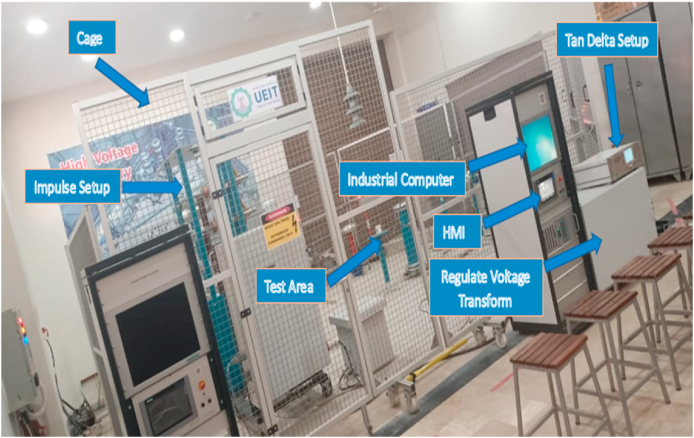
Front view of high voltage LAB.

**Fig. 6:**
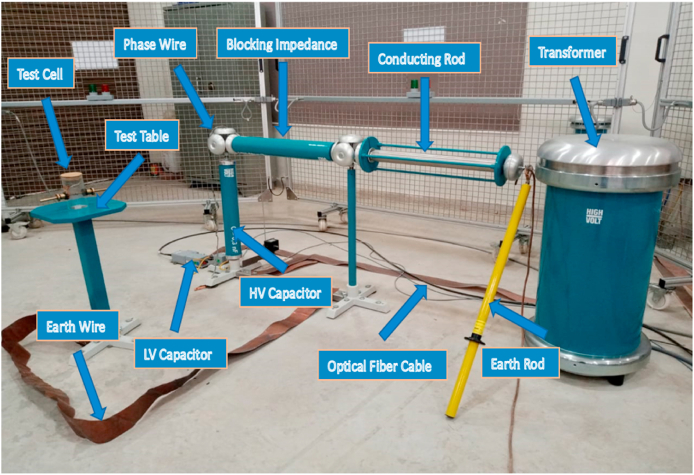
Experimental setup of AC BDV.

In accordance with the IEC 60156 standard method as shown in Table 3, the break-down voltage measurements were carried out using a High Voltage setup designed by High Volt, Germany, in Department of Electrical Engineering, KFUEIT as shown in Fig. 5, consisting of an oil test cell with a 300 mL capacity, an adjustable electrode system, and a high voltage generator capable of reaching up to 100 kV RMS (50 Hz). The break-down voltage test was performed using a mushroom-mushroom type electrode having of 12.5 mm diameter and a spacing with 2.5 mm, with a voltage increment of 2 kV/s until breakdown occurred as shown in Fig. 6.

**Table 3:** Comparison of ASTM and IEC standards for measuring AC.

Standards	ASTM D1816	ASTM D877	IEC 60156
Description	Mostly widely used standard in North America	Old standard	Various countries have adopted it
Shape of electrode			
Electrode material	Polished Brass	Brass	Brass/Bronze/Stainless Steel
Size of electrode	Diameter: 36mm	Diameter: 25.4 mm Thickness:≥3.18 mm Sharp edge radius:≤0.254 mm	Diameter: 12.5 mm–13 mm
Electrode gap	2mm/1 mm	Thickness:≥3.18 mm	2.5 mm
Voltage rate of rising	0.5 kV/s	Sharp edge radius:≤0.254 mm	2 kV/s
Time between breakdowns	1 to 1.5 minutes	2.54 mm	2 min
Stirring	Continuous with impeller (200–300 rpm)	3 kV/s	Optional with a magnetic bar
Breakdown Value	Mean of 5 measurements	1 minutes	Mean of 6 measurements

The BDV test was also conducted for nanofluids in which the BDV occurred for an electrode gap of 2.5 mm (i.e., SiO_2_, TiO_2_, Fe_2_O_3_, graphene, and Al_2_O_3_). Six measurements were performed, giving 6 points that were sufficient for statistical analysis. The AC breakdown voltage data for 2.5 mm electrode gaps were analyzed for conformity to extreme value, Weibull, and normal distributions using Anderson-Darling statistics. The normal and EV distributions were used to determine the voltages corresponding to 1%, 10%, and 50% risk levels.

## 3 Results

### 3.1 AC BDV test

The average AC breakdown voltage measurements of the nanofluids were compared to average AC BDV of cottonseed oil (CSO), and the results indicated a significant increase in the AC BDV of nanofluids compared with pure cottonseed oil (CSO). The addition of nanoparticles did not have a negative effect on the AC BDV, as shown in Table 4.

**Table 4:** This mean AC BDV & Electric Field Strength (EFS) of Cottonseed Oil, Mineral Oil & Nanofluids.

Nanoparticles	Concentration (g/ml %)	Mean BDV (KV)	Increment (KV)	EFS (KV/mm)
**SiO**_**2**_	0.025	42.2	14.76	16.88
	0.05	78	34.7	31.2
	0.075	51.2	23.76	20.48
**TiO**_**2**_	0.025	41.3	13.86	16.52
	0.05	75	36.1	30
	0.075	52.6	25.16	21.04
**Al**_**2**_**O**_**3**_	0.025	39.6	12.16	15.84
	0.05	80	33.8	32
	0.075	54.3	26.86	21.72
**Fe**_**2**_**O**_**3**_	0.025	38.8	11.36	15.52
	0.05	53.2	15.86	21.28
	0.075	52.1	24.66	20.84
**Graphene**	0.025	43.8	16.36	17.52
	0.05	72	37.6	28.8
	0.075	57.8	30.36	23.12
**Mineral Oil**	–	48	–	19.2

Nanofluids of cottonseed oil with Al_2_O_3_, TiO_2_, Fe_2_O_3_, SiO_2_, and graphene nanoparticles were prepared using a simple one-step mixing process at a 0.025 wt/vol% concentration. The AC breakdown voltage test was performed on each nanofluid, and the results indicate that breakdown voltage of the cottonseed oil was increased with addition of nanoparticles. The breakdown voltages for the nanofluids containing Al_2_O_3_, TiO_2_, Fe_2_O_3_, SiO_2_, and graphene were measured at 39.6 kV, 41.3 kV, 38.8 kV, 42.2 kV, and 43.8 kV, respectively. These findings highlight graphene nanofluids as exhibiting the most substantial enhancement in breakdown voltage compared to other counterparts.

In the case of a 0.05 wt/vol% concentration, nanofluids of cottonseed oil containing Al_2_O_3_, TiO_2_, Fe_2_O_3_, SiO_2_, and graphene nanoparticles were prepared through a simple mixing process. The AC breakdown voltage test results revealed a significant impact of nanoparticle addition on the breakdown voltage of nanofluids. Specifically, the breakdown voltages for nanofluids with Al_2_O_3_, TiO_2_, Fe_2_O_3_, SiO_2_, and graphene nanoparticles were determined as 80 KV, 75 KV, 53.2 KV, 78 KV, and 72 KV, respectively. These outcomes underscore the substantial enhancement in AC breakdown voltage achieved by the addition of nanoparticles, with Al_2_O_3_ nanoparticles exhibiting the highest improvement among the tested nanoparticles. These results suggest that nanofluids incorporating graphene nanoparticles at a 0.05 wt/vol% concentration hold promise as insulation materials for high-voltage applications.

At a concentration of 0.075 wt/vol%, the Al_2_O_3_ nanofluid exhibited an AC breakdown voltage of 54.3 kV, signifying a notable increase compared to the base fluid. The TiO_2_ nanofluid at the same concentration demonstrated an AC breakdown voltage of 52.6 kV, surpassing that of the base fluid, indicating the potential of TiO_2_ nanoparticles as effective insulating materials. The Fe_2_O_3_ nanofluid at 0.075 wt/vol% concentration displayed an AC breakdown voltage of 52.1 kV, suggesting an improvement in electrical strength relative to the base fluid. The SiO_2_ nanofluid at the same concentration exhibited an AC breakdown voltage of 51.2 kV, indicating an enhancement in the electrical strength of the nanofluid. Notably, the graphene nanofluid at 0.075 wt/vol% concentration showcased the highest AC breakdown voltage at 57.8 kV, significantly surpassing that of the base fluid. This underscores the excellent potential of graphene nanoparticles as insulating materials.

Overall, the AC breakdown voltage test results underscore that the addition of nanoparticles at a 0.075 wt/vol% concentration markedly enhances the electrical strength of cottonseed oil-based nanofluids, with graphene nanoparticles exhibiting the most promising outcomes. The BDV, or breakdown voltage, of an insulator, is the voltage at which it starts to conduct electricity, creating a channel of conductivity within the insulating material un-der specific electrical stress. This value represents the dielectric strength of electrical insulation and may vary depending on the testing protocol used [46]. Table 4 presents the mean AC breakdown voltage and percentage increase (%) of both synthetic ester and nanofluids. While Fig. 8 from (a) to (0) shows the AC BDV results on HMI respectively and their comparison result shown in Fig. 9. Additionally, the corresponding break-down electric field strength (EFS) is provided in Table 4, calculated using the formula:

(1) E=V/d

where E represents the electric field strength, V is the applied voltage, and d is the distance between the two electrodes (which was set to 2.5 mm for this study).

**Fig. 7:**
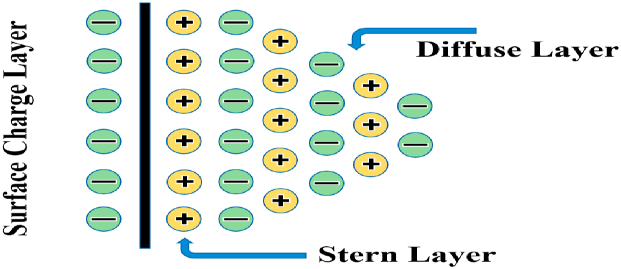
Formation of electric double layer (EDL) [33].

**Fig. 8:**
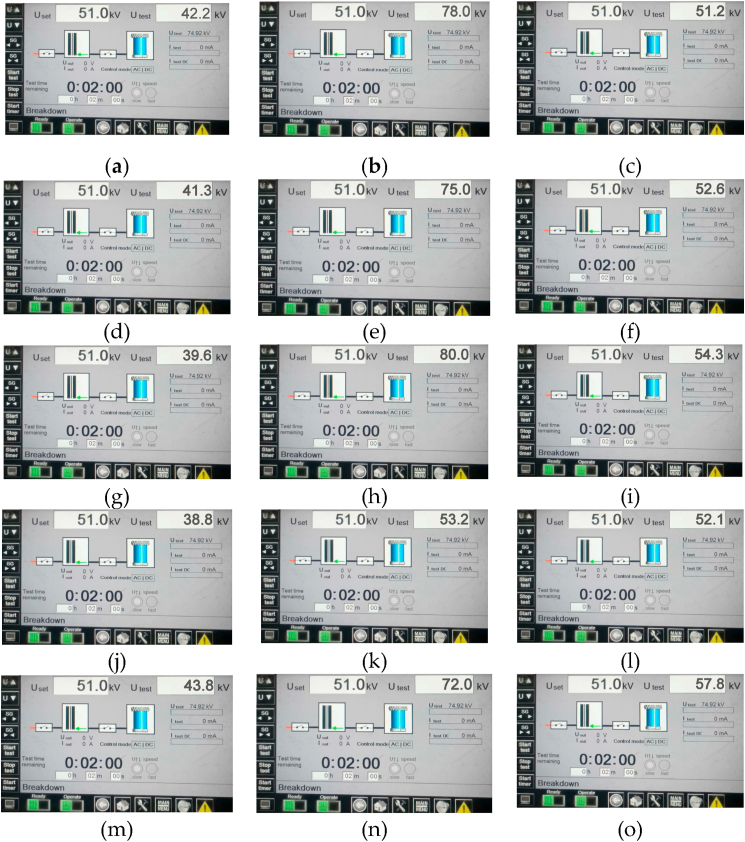
Experimental AC BDV Results of all samples, in which (a),(b) & (c) shows the results of SiO_2_ at 0.025, 0.05 and 0.075 respectively, (d),(e) & (f) shows the results of TiO_2_ at 0.025, 0.05 and 0.075 respectively, (g),(h) & (i) shows the results of Al_2_O_3_ respectively, (j),(k) & (l) shows the results of Fe_2_O_3_ at 0.025, 0.05 and 0.075 respectively and (m),(n) & (o) shows the results of graphene at 0.025, 0.05 and 0.075 respectively.

**Fig. 9:**
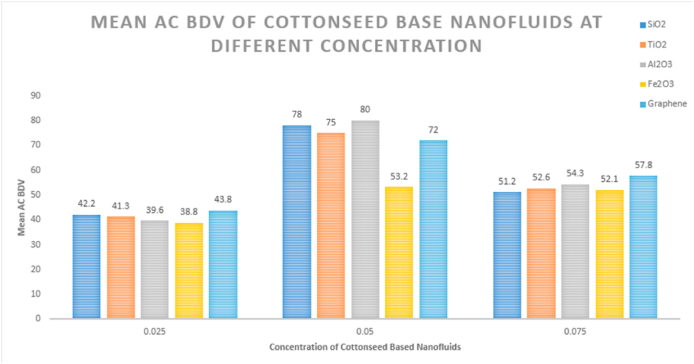
Comparison of Mean AC BDV of all concentration of cottonseed based nanofluids.

When a high voltage is applied to insulating oil, it can create a conducting channel between two electrodes, allowing charge carriers to move quickly towards the opposite electrode, resulting in a streamer and electrical breakdown. The formation of streamers in insulating oil is primarily influenced by the applied high voltage and electric field strength. However, the behavior of resulting nanofluids changes when the nanomaterials are scatter in insulating oil due to alterations in streamer electrodynamics within the medium. The presence of nanomaterials in the insulating oil forms a surface that attracts fast moving charge carriers, which leads to delays in relaxation time & streamer propagation. As a result, the BDV strength of the insulating oil based nanofluids is improved [[47], [48], [49]]. The interaction between nanoparticles and the insulating oil forms a strong layer called the Stern layer, and the diffuse layer is formed around this layer. This process attracts more charge carriers until it reaches saturation, thereby creating an electrical double layer around the particles. [50,51], which is dependent on several factors affect these layers, such as high specific surface area of nanoparticles, high bandgap, differences in the electrical conductivity & dielectric constant of nanoparticles and insulating oil, and the sur-face energy states, which refers to the proximity of oxygen vacancy [[52], [53], [54], [55]].

The difference in electrical properties between the insulating oil and nanomaterial causes a non-continuous interface to form, as the positions of their valence and conduction bands are different. This results in an electronic band that bends towards the inter-face, inducing surface energy states. These energy states often include defect states, like interstitial or chemical defects, that are present on the surface of the nanomaterials. As a result of the dispersion of nanomaterials in the insulating oil, these defect states affect charge density, leading to attraction of charges from insulating medium to surface of nanoparticles, which forms an electrical double layer on their surface. In this study, the improved AC BDV of nanofluids could be attributed to the process of charge trapping & de-trapping, which occurred because to formation of an electrical double layer on surface of nanoparticles in insulating medium, as illustrated in Fig. 7.

When the size of nanoparticles decreases, their specific surface area increases, leading to an increase in surface energy and binding energy [56,57]. Nanoparticles with a large specific surface area can generate electrical double layer on the liquid solid interface when dispersed in insulating oil [[58], [59], [60]]. However, studies have shown that dispersion of the nanoparticles with wide bandgap in an insulating oil can capture more electrons due to fewer repulsive forces from the electron population in valence band [61]. This results in a delay in streamer propagation and an increase in breakdown voltage [61]. Due to the increase in charge density on the surface of the nanomaterials over time, a saturation point was eventually reached. Once all available sites on the nanoparticle surface were occupied, further incoming charge carriers were repelled, resulting in the release of previously trapped free charges that then moved towards the opposite electrode, ultimately causing a breakdown in insulating oil medium. Therefore, the high specific surface area & wide bandgap of the nanomaterials resulted in the formation of a robust electrical double layer on their surface, leading to an improved AC dielectric strength of the nanofluids.

### 3.2 Physical chemical properties test of nanofluids

We perform these tests on transformer oil to ensure that it meets the required physical and chemical properties for safe and reliable operation of high voltages. These properties include the flash point, fire point, pour point, cloud point, viscosity, acidity, moisture con-tent, resistivity, and dissipation factor. Each of these characteristics plays a vital role in assessing the effectiveness of the oil under various circumstances. For instance, the flash point and fire point are instrumental in determining the oil's safety by preventing potential fire hazards during operation. Similarly, the pour point and cloud point influence the oil's performance in cold climates by determining its ability to flow through the transformer's components. The viscosity of the oil impacts its circulation within the transformer, while acidity and moisture content can affect its chemical stability and overall performance. Monitoring these properties allows for the early detection of any potential issues or problems with the transformer oil, helping to prevent damage or safety hazards.

The flash point and fire point are particularly important physical and chemical properties in ensuring the safety of insulating oil in high voltages. The flash point represents the minimum temperature at which the oil vapor can ignite when exposed to a flame or spark, while the fire point is the minimum temperature at which the oil will continue to burn after ignition. Maintaining adequate levels of these properties is crucial to prevent the oil from catching fire during normal transformer operation, thus averting potential damage or destruction of equipment. Therefore, it is essential to assess the flash and fire points of cottonseed oil when considering its use as insulating oil in high voltages.

The pour point and cloud point are two significant physical properties that play a crucial role in assessing the performance of insulating oils under cold conditions. The pour point signifies the temperature at which the oil's viscosity becomes too high for proper flow, while the cloud point marks the temperature at which wax crystals start to form within the oil, resulting in cloudiness. Both attributes can impact the oil's ability to circulate through the transformer's components, potentially leading to operational issues during cold weather. Excessive viscosity may hinder effective circulation, posing a risk of transformer damage. Likewise, the presence of wax crystals could obstruct transformer components, causing operational disruptions.

Hence, it is crucial to assess the pour point and cloud point of cottonseed oil when contemplating its application as an insulating oil in high-voltage environments. Furthermore, viscosity stands out as another pivotal physical property essential for gauging the efficacy of insulating oils in high-voltage settings. Viscosity denotes the oil's resistance to flow and serves as a critical determinant of its ability to circulate through the transformer's components smoothly. An oil with high viscosity may encounter challenges in circulation, potentially causing operational issues and posing a risk of transformer damage. Conversely, a low-viscosity oil may fail to offer adequate lubrication, resulting in heightened wear and tear on the transformer's components. Hence, meticulous consideration of the viscosity of cottonseed oil is imperative when choosing an insulating oil for high-voltage applications.

Acidity represents a significant chemical attribute, indicating the presence of free fatty acids in the oil. Elevated acidity levels can result in heightened corrosion and deterioration of transformer components over time, ultimately diminishing the transformer's lifespan and amplifying the risk of operational issues. Hence, it is imperative to assess the acidity of cottonseed oil when contemplating its application as an insulating oil in high-voltage scenarios.

Similarly, moisture content emerges as another crucial chemical characteristic to scrutinize when assessing the suitability of cottonseed oil as an insulating oil in high-voltage settings. Even minimal moisture levels can substantially reduce the oil's breakdown voltage, potentially leading to operational challenges within the transformer. Moisture also fosters the proliferation of microorganisms, further deteriorating the oil and compromising its efficacy as an insulator. Consequently, thorough evaluation of cottonseed oil's moisture content is indispensable when considering its utilization as an insulating oil in high-voltage applications.

Furthermore, resistivity stands out as a critical electrical property governing the oil's insulation capability against electrical charges. Inadequately low resistivity levels may undermine the oil's effectiveness in insulating the transformer against electrical charges, thereby predisposing the equipment to operational issues and potential damage. Thus, evaluating the resistivity of cottonseed oil is pivotal in assessing its suitability as an insulating oil in high-voltage contexts.

The dissipation factor stands as another crucial physical property of transformer oil closely associated with its dielectric strength. It is defined as the ratio of the power dissipated in the oil to the power transmitted through it. This parameter is determined by subjecting a sample of the oil to high-frequency voltage and measuring the resulting current flow. A high dissipation factor indicates that the oil is losing energy to its surroundings, potentially leading to overheating and subsequent transformer damage. Therefore, assessing the dissipation factor of transformer oil is essential to ensure it offers sufficient insulation without causing operational issues. Evaluation of the physical and chemical properties of transformer oil is imperative for ensuring the safe and reliable operation of high voltages. Parameters such as fire point and flash point determine the oil's safety, while pour point and cloud point influence its performance in cold conditions. Viscosity dictates the oil's circulation through transformer components. Additionally, acidity, moisture content, resistivity, and dissipation factors are critical factors that can affect the oil's suitability for high-voltage applications. By consistently measuring and monitoring these properties, it is feasible to ascertain that transformer oil provides adequate insulation and does not pose operational challenges.

**Flash & Fire Point Test.** To find the flash and fire points of the cottonseed oil prepared with nanoparticles, standard tests were also followed. The tests were conducted using an open-cup apparatus followed by ASTM D93 standard method, which is a common method for determining the flash and fire points of liquids.

**Fig. 10:**
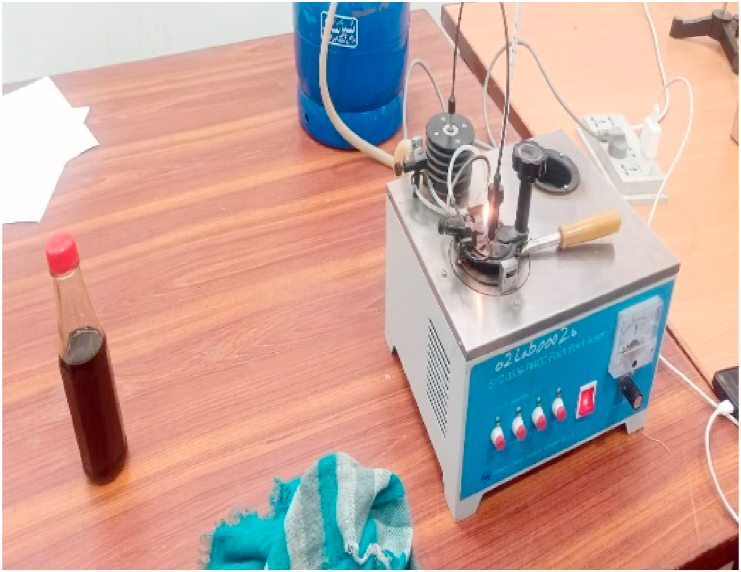
The experiment setup shows the flash point.

For the flash point test, a small amount of the cottonseed oil was placed in the open-cup apparatus and a small flame was brought near the surface of the oil at regular intervals. The temperature of the oil was gradually increased until the vapor above the oil ignited, producing a flash. The temperature at which the flash occurred was recorded as the flash point shown in Fig. 10.

**Fig. 11:**
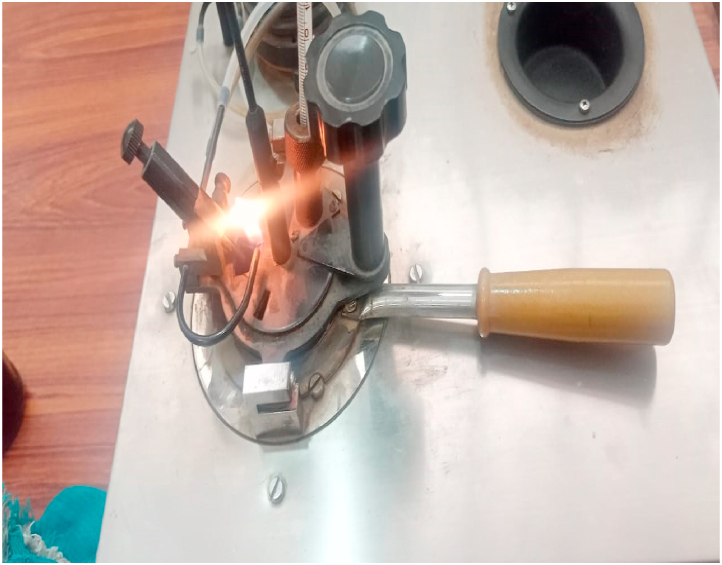
The experimental setup shows the fire point.

For the fire point test, the temperature was further increased until the oil caught fire and continued to burn for at least 5 s. The temperature at which the oil caught fire and continued to burn was recorded as the fire point shown in Fig. 11.

Initially, fire and flash point assessments were conducted on cottonseed oil infused with nanoparticles of Al_2_O_3_, TiO_2_, Fe_2_O_3_, SiO_2_, and graphene at a concentration of 0.025 wt/vol%. The incorporation of these nanoparticles into the oil demonstrated enhancements in thermal stability and a reduction in flammability, consequently elevating both fire and flash points. Specifically, the fire point and flash point of cottonseed oil augmented with 0.025 wt/vol% Al_2_O_3_ nanoparticles were determined to be 250 °C and 140 °C, respectively, marking a 5% and 10% escalation from the base oil. TiO_2_ nanoparticles exhibited an increase of 7% and 14%, respectively, compared to the base oil, showcasing fire and flash points of 255 °C and 145 °C. The inclusion of Fe_2_O_3_ nanoparticles led to a rise of 10% and 20%, respectively, over the base oil, with fire and flash points reaching 265 °C and 150 °C. SiO_2_ nanoparticles yielded a 6% and 12% increase, respectively, compared to the base oil, resulting in fire and flash points of 252 °C and 142 °C. Notably, graphene nanoparticles demonstrated the most significant increase, with fire and flash points rising by 8% and 24%, respectively, compared to the base oil, registering values of 260 °C and 155 °C. These findings underscore the potential of nanoparticle integration for enhancing fire resistance and thermal stability in high-voltage and high-temperature settings.

Subsequently, fire and flash point evaluations were conducted on cottonseed oil infused with nanoparticles of Al_2_O_3_, TiO_2_, Fe_2_O_3_, SiO_2_, and graphene at a concentration of 0.05 wt/vol%. The incorporation of these nanoparticles into the oil resulted in enhanced thermal stability and reduced flammability, consequently elevating both fire and flash points. Specifically, the fire point and flash point of cottonseed oil augmented with 0.05 wt/vol% Al_2_O_3_ nanoparticles were determined to be 253 °C and 143 °C, respectively, marking a 6% and 13% escalation from the base oil. TiO_2_ nanoparticles exhibited an increase of 10% and 20%, respectively, over the base oil, with fire and flash points reaching 260 °C and 150 °C. The inclusion of Fe_2_O_3_ nanoparticles led to a rise of 15% and 30%, respectively, compared to the base oil, with fire and flash points registering at 272 °C and 158 °C. SiO_2_ nanoparticles resulted in a 7% and 15% increase, respectively, over the base oil, yielding fire and flash points of 256 °C and 146 °C. Remarkably, graphene nanoparticles demonstrated the most substantial increase, with fire and flash points rising by 12% and 40%, respectively, compared to the base oil, recording values of 256 °C and 170 °C. These findings further underscore the potential of nanoparticle integration for enhancing fire resistance and thermal stability in high-voltage and high-temperature applications.

Following that, fire and flash point assessments were conducted on cottonseed oil containing Al_2_O_3_, TiO_2_, Fe_2_O_3_, SiO_2_, and graphene nanoparticles at a concentration of 0.075 wt/vol%. The incorporation of these nanoparticles into the oil resulted in enhanced thermal stability and reduced flammability, consequently leading to increased fire and flash points. Specifically, cottonseed oil with 0.075 wt/vol% Al_2_O_3_ nanoparticles exhibited fire and flash points of 255 °C and 147 °C, respectively, marking an 8% and 16% increase compared to the base oil. TiO_2_ nanoparticles demonstrated a 12% and 24% increase, respectively, over the base oil, yielding fire and flash points of 263 °C and 155 °C. Fe_2_O_3_ nanoparticles showed a remarkable increase of 19% and 40%, respectively, compared to the base oil, resulting in fire and flash points of 276 °C and 167 °C. SiO_2_ nanoparticles demonstrated a 9% and 19% increase, respectively, compared to the base oil, with fire and flash points of 258 °C and 151 °C.

Additionally, graphene nanoparticles exhibited the highest increase, with fire and flash points rising by 14% and 52%, respectively, compared to the base oil, registering values of 268 °C and 183 °C. These findings further underline the potential of utilizing these nanoparticles to enhance fire resistance and thermal stability in high-voltage and high-temperature applications. The flash and fire point tests were conducted in triplicate, and the average values were determined, as presented in Table 5. These results indicate that the cottonseed oil prepared with nanoparticles has a high flash and fire point, which makes it a safe option for use as insulating oil in high voltages. Different concentrations show that as it increases from 0.025% to 0.075% the Flash and Fire point also increase. The high flash and fire points ensure that the oil will not ignite or continue to burn during operation, reducing the risk of fire and ensuring safe and efficient operation of transformer.

**Table 5:** Comparison between flash & fire points of nanofluids with all concentration & MO.

NP	Concentration (g/ml %)	Mean Flash Point (^0^C)	Increment (^0^C)	Mean Fire Point (^0^C)	Increment (^0^C)
SiO_2_	0.025	242	27	252	2
	0.05	246	31	256	6
	0.075	251	36	258	8
TiO_2_	0.025	245	30	255	5
	0.05	250	35	260	10
	0.075	255	40	263	13
Al_2_O_3_	0.025	240	25	250	0
	0.05	243	28	253	3
	0.075	247	32	255	5
Fe_2_O_3_	0.025	250	35	265	15
	0.05	258	43	272	17
	0.075	267	52	276	21
Graphene	0.025	255	40	260	10
	0.05	270	55	265	15
	0.075	283	68	268	18
Mineral Oil	–	100–175	–	110–185	–

**Cloud & Pour Point Test.** To determine the pour and cloud points of the cottonseed oil prepared with nanoparticles, standard test procedures were followed. The tests were conducted using a cooling bath and a cloud and pour point apparatus, which is a common method for determining the pour and cloud points of petroleum products.

**Fig. 12:**
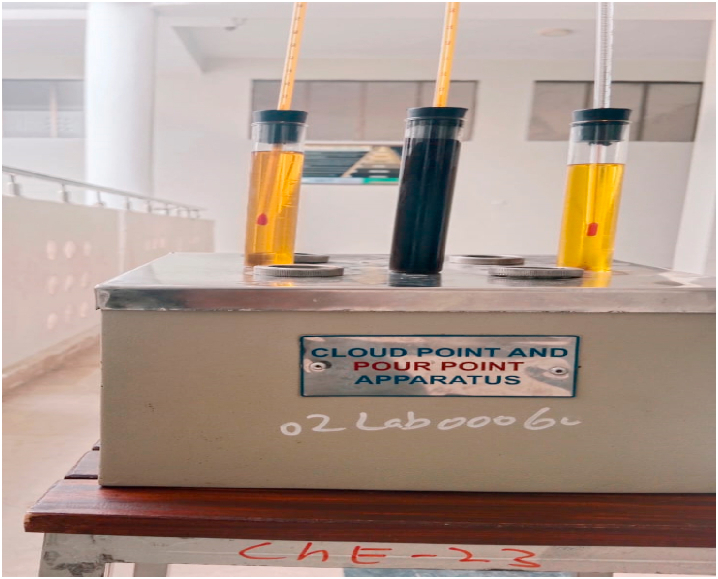
Experimental setup of cloud and pour point.

For the pour point test, a small amount of the cottonseed oil was placed in a test tube and placed in a cooling bath. The temperature of the cooling bath was gradually de-creased until the oil was too viscous to flow. The temperature at which the oil became too viscous to flow was recorded as the pour point shown in Fig. 12.

For the cloud point test, a small amount of the cottonseed oil was placed in a test tube and placed in a cooling bath. The temperature of the cooling bath was gradually de-creased until wax crystals began to form in the oil, causing it to become cloudy. The temperature at which the oil became cloudy was recorded as the cloud point shown in Fig. 12.

The incorporation of 0.025 wt/vol% Al_2_O_3_, TiO_2_, Fe_2_O_3_, SiO_2_, and graphene nanoparticles into cottonseed oil yielded notable enhancements in its low-temperature characteristics, evidenced by reductions in both Pour Point and Cloud Point temperatures. In comparison to the base oil, the Al_2_O_3_ nanofluid exhibited lowered Pour Point and Cloud Point temperatures by 3 °C and 5 °C, respectively, whereas those of the TiO2 nanofluid decreased by 4 °C and 7 °C. Similarly, the Fe_2_O_3_ nanofluid demonstrated a 2 °C reduction in both Pour Point and Cloud Point temperatures, while the SiO2 nanofluid showed reductions of 3 °C and 6 °C, respectively. Notably, the graphene nanofluid displayed the most significant effect, with reductions of 4 °C and 8 °C in Pour Point and Cloud Point temperatures, respectively. Furthermore, the nanofluids mitigated the formation of haze or cloudiness, underscoring their potential application in lubrication in cold and extremely cold environments.

Likewise, the Pour Point and Cloud Point of cottonseed oil were examined following the addition of 0.05 wt/vol% of Al_2_O_3_, TiO_2_, Fe_2_O_3_, SiO_2_, and graphene nanoparticles. With Al_2_O_3_ nanoparticles, the Pour Point and Cloud Point of cottonseed oil decreased by 4 °C and 2 °C, respectively. For TiO_2_ nanoparticles, there was a decrease of 6 °C and 4 °C, respectively, in Pour Point and Cloud Point temperatures. Similarly, Fe_2_O_3_ nanoparticles led to reductions of 9 °C and 6 °C, respectively, in Pour Point and Cloud Point temperatures. For SiO_2_ nanoparticles, reductions of 5 °C and 3 °C were observed in Pour Point and Cloud Point temperatures, respectively. Lastly, with graphene nanoparticles, reductions of 8 °C and 8 °C were noted in Pour Point and Cloud Point temperatures, respectively. The incorporation of all nanoparticles resulted in significant improvements in low-temperature flow properties and decreased cloudiness of cottonseed oil, suggesting their potential application in cold weather conditions.

Similarly, these represent the low-temperature performance outcomes of various nanoparticle types incorporated into cottonseed oil. With the addition of 0.075 wt/vol% Al_2_O_3_ nanoparticles to the oil, there was a 2 °C improvement in Pour Point and a 5 °C improvement in Cloud Point. Likewise, the inclusion of 0.075 wt/vol% TiO2 nanoparticles resulted in reductions of 5 °C and 8 °C in Pour Point and Cloud Point, respectively. Furthermore, the addition of 0.075 wt/vol% Fe_2_O_3_ nanoparticles led to enhancements of 8 °C and 10 °C in Pour Point and Cloud Point, respectively. Similarly, the incorporation of 0.075 wt/vol% SiO_2_ nanoparticles improved the Pour Point and Cloud Point by 3 °C and 7 °C, respectively. Finally, with the inclusion of 0.075 wt/vol% graphene nanoparticles, there were improvements of 6 °C and 9 °C in Pour Point and Cloud Point, respectively. Overall, all nanoparticle variants augmented the oil's low-temperature performance and prevented the occurrence of cloudiness or haze, thus positioning them as potential candidates for use in cold weather applications.

The pour and cloud point tests were conducted in triplicate, and the average values were calculated as illustrate in Table 6. These results show that the cottonseed oil pre-pared with nanoparticles has a low pour and cloud point, which makes it suitable for use as insulating oil in high voltages operating at low temperatures. The low pour point ensures that the oil will remain fluid and able to flow through the transformer's components, while the low cloud point indicates that the oil will not form wax crystals that could obstruct the flow of oil or cause operational problems. At a concentration of 0.025 wt/vol%, the nanofluids containing Al_2_O_3_, TiO_2_, and SiO_2_ exhibited pour points lower than those of the base oil, whereas the Fe_2_O_3_ and graphene nanofluids demonstrated slightly elevated pour points. Additionally, all nanofluids displayed cloud points slightly above that of the base oil. At a concentration of 0.05 wt/vol%, the Al_2_O_3_ and Fe_2_O_3_ nanofluids exhibited lower pour points compared to the base oil, while the TiO_2_, SiO_2_, and graphene nanofluids displayed slightly higher pour points. Similar to the previous concentration, the cloud points for all nanofluids were marginally higher than that of the base oil. Moving to a concentration of 0.075 wt/vol%, the Al_2_O_3_, TiO_2_, and Fe_2_O_3_ nanofluids showcased lower pour points than the base oil, whereas the SiO_2_ and graphene nanofluids exhibited slightly higher pour points. Once again, the cloud points for all nanofluids were slightly higher than those of cottonseed oil. These findings underscore the influence of nanoparticles on the pour and cloud points of the oil, emphasizing the role of nanoparticle concentration in determining their impact, as outlined in Table 6.

**Table 6:** Comparison between pour & cloud points of nanofluids with all concentration.

NP	Concentration (g/ml %)	Mean Pour Point (^0^C)	Decrement (^0^C)	Mean Cloud Point (^0^C)	Decrement (^0^C)
SiO_2_	0.025	−2	3	14	6
	0.05	−11	5	23	3
	0.075	−13	3	18	7
TiO_2_	0.025	−1	4	11	7
	0.05	−12	6	22	4
	0.075	−15	5	17	8
Al_2_O_3_	0.025	−2	3	13	5
	0.05	−10	4	24	2
	0.075	−12	2	20	5
Fe_2_O_3_	0.025	−3	2	12	4
	0.05	−15	9	20	6
	0.075	−18	8	15	10
Graphene	0.025	−1	4	8	10
	0.05	−14	8	18	8
	0.075	−16	6	16	9

#### 3.2.1 Viscosity test

**Fig. 13:**
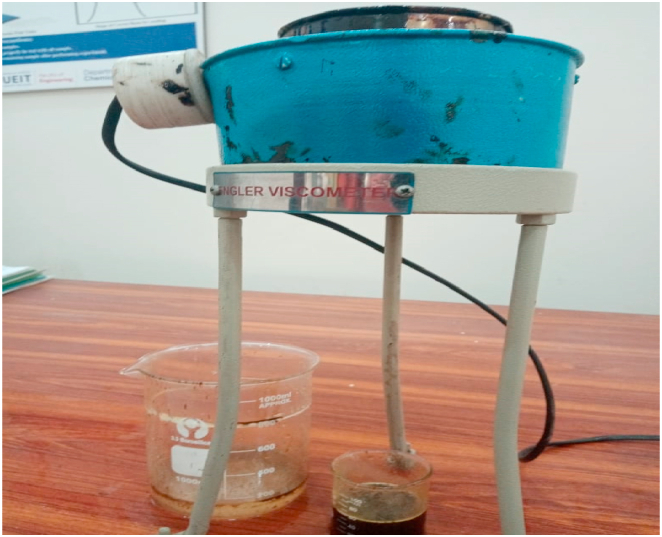
Experimental setup of viscosity test.

To determine the viscosity of the cottonseed oil prepared with nanoparticles, standard test procedures were followed. The test was conducted using a viscometer, which is a common method for measuring the viscosity of liquids. A small amount of the cottonseed oil was placed in the viscometer, and the viscometer was set to a specific speed. The oil was allowed to flow through the viscometer, and the time it took to flow through a specific distance was recorded. The experimental setup shown in Fig. 13. The viscosity of the oil was calculated using the formula:

Viscosity=(TimexCalibrationFactor)/Volume

Firstly, have nanofluids of cottonseed oil with Al_2_O_3_, TiO_2_, Fe_2_O_3_, SiO_2_, and graphene nanoparticles at a 0.025 wt/vol% concentration, and I performed a “viscosity test” of cottonseed oil with every nanoparticle at a 0.025 concentration. Addition of Al_2_O_3_ nanoparticles to oil increased its viscosity from 35 cSt (base oil) to 31.95 cSt, TiO_2_ nanoparticles in-creased the viscosity from 35 cSt to 31.79 cSt, Fe_2_O_3_ nanoparticles increased the viscosity from 35 cSt to 32.14 cSt, SiO_2_ nanoparticles increased the viscosity from 35 cSt to 31.95 cSt, and graphene nanoparticles increased the viscosity from 35 cSt to 33.05 cSt. The results show that the addition of nanoparticles to the cottonseed oil increases its viscosity, with graphene nanoparticles having the most significant effect on viscosity.

Then conducted a viscosity test on nanofluids of cottonseed oil with Al_2_O_3_, TiO_2_, Fe_2_O_3_, SiO_2_, and graphene nanoparticles at a 0.05 wt/vol% concentration. The viscosity of the base oil was found to be 35 cSt. After the addition of 0.05 wt/vol% Al_2_O_3_ nanoparticles, the viscosity increased to 57.5 cSt. Similarly, the addition of 0.05 wt/vol% TiO_2_ nanoparticles resulted in a viscosity increased 52.2 cSt. The Fe_2_O_3_ nanoparticles caused the most significant viscosity increase 48.6 cSt. The addition of SiO_2_ nanoparticles resulted in a 20% increase in viscosity to 53.4 cSt, while graphene nanoparticles caused a viscosity increase 50.9 cSt. These results indicate that addition of these nanoparticles at 0.05 wt/vol% con-centration can significantly increase the viscosity of cottonseed oil, making it potentially useful for various industrial applications.

Lastly, the viscosity test was conducted on nanofluids prepared using cottonseed oil as the base oil and Al_2_O_3_, TiO_2_, Fe_2_O_3_, SiO_2_, and graphene nanoparticles at a 0.075 wt/vol% concentration. The results showed that addition of nanoparticles increased viscosity of the nanofluids compared to base oil. The viscosity of the Al_2_O_3_ nanofluid was 43.2 cSt, the TiO_2_ nanofluid was 42.6 cSt, the Fe_2_O_3_ nanofluid was 44.1 cSt, the SiO_2_ nanofluid was 42.9 cSt, and the graphene nanofluid was 45.2 cSt. These results suggest that addition of nanoparticles at 0.075 wt/vol% concentration can significantly alter the viscosity of cottonseed oil, which may have implications for their potential use in various applications.

The viscosity test was conducted in triplicate, and the average value was calculated. Overall, the results indicate that the addition of nanoparticles to cottonseed oil resulted in a decrease in viscosity, and extent of decrease increased with increase in nanoparticle concentration. Among the nanoparticles studied, graphene nanoparticles caused the most significant reduction in viscosity, followed by Al_2_O_3_, SiO_2_, TiO_2_, and Fe_2_O_3_ nanoparticles, respectively as shown in Table 7. These results indicate that the cottonseed oil prepared with nanoparticles has a moderate viscosity, which makes it suitable for use as insulating oil in high voltages. The viscosity of the oil ensures that it will flow properly through the transformer's components, while also providing enough resistance to flow to lubricate and protect the components. The moderate viscosity also indicates that the oil is less likely to degrade or break down under the high temperatures and stresses present in the *trans*-former, ensuring that it will provide reliable and long-lasting performance.

**Table 7:** Comparison between viscosity of nanofluids with all concentration.

NP	Concentration (g/ml %)	Viscosity (cSt)	Increment (cSt)
SiO_2_	0.025	31.95	3.05
	0.05	53.4	18.4
	0.075	42.9	7.9
TiO_2_	0.025	31.79	3.21
	0.05	52.2	17.2
	0.075	42.6	7.6
Al_2_O_3_	0.025	31.95	3.05
	0.05	57.5	22.5
	0.075	43.2	8.2
Fe_2_O_3_	0.025	32.14	2.86
	0.05	48.6	13.6
	0.075	44.1	9.1
Graphene	0.025	33.05	1.95
	0.05	50.9	15.9
	0.075	45.2	10.2

**Acidity Test.** To assess the acidity of cottonseed oil infused with nanoparticles, standard test procedures were implemented. The evaluation utilized an acid-base titration method, a widely employed technique for measuring acidity in oils and fats.

**Fig. 14:**
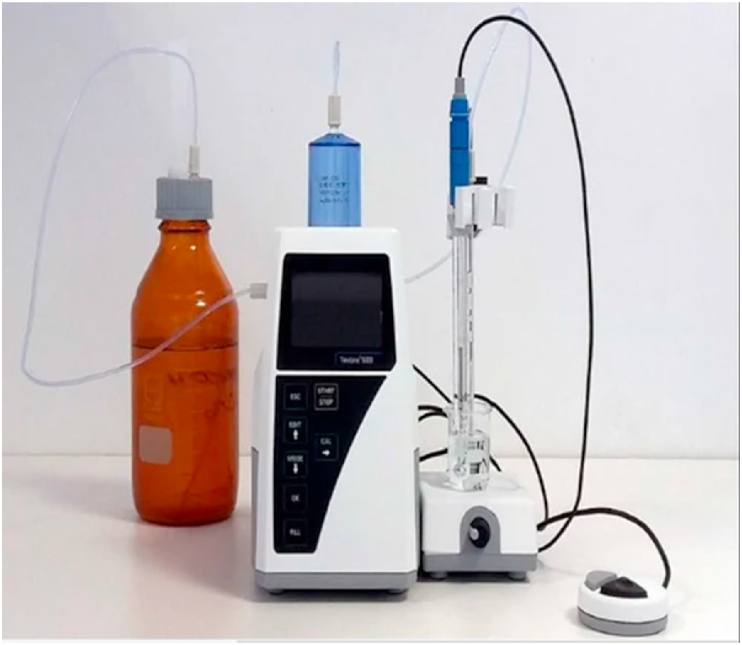
Experimental setup of acidity test.

In this procedure, a sample of cottonseed oil was dissolved in an organic solvent, followed by the addition of a small quantity of indicator solution to the mixture. Subsequently, a standard solution of potassium hydroxide (KOH) was gradually introduced to the mixture until the indicator's color changed, indicating neutralization of all free acids in the oil. The volume of KOH solution required for neutralization was measured, as depicted in Fig. 14, and the oil's acidity was calculated using the formula:

Acidity = (Volume of KOH Solution × Normality of KOH Solution × 56.1)/Weight of Sample.

Initially, an acidity test was conducted on nanofluids of cottonseed oil containing Al_2_O_3_, TiO_2_, Fe_2_O_3_, SiO_2_, and graphene nanoparticles at a concentration of 0.025 wt/vol%. The results revealed that the nanofluid with Al2O3 nanoparticles exhibited the highest acidity level of 0.45 mg KOH/g, while the nanofluid containing graphene nanoparticles displayed the lowest acidity level of 0.31 mg KOH/g. Additionally, nanofluids containing TiO_2_, Fe_2_O_3_, and SiO_2_ nanoparticles showcased acidity levels of 0.36 mg KOH/g, 0.39 mg KOH/g, and 0.37 mg KOH/g, respectively. These findings indicate that the addition of nanoparticles influences the acidity level of cottonseed oil, with Al_2_O_3_ nanoparticles exerting the most significant impact and graphene nanoparticles having the least impact. Considering these acidity levels is crucial when contemplating the practical applications of these nanofluids.

Subsequently, nanofluids consisting of cottonseed oil with Al_2_O_3_, TiO_2_, Fe_2_O_3_, SiO_2_, and graphene nanoparticles at a concentration of 0.05 wt/vol% underwent acidity testing. The acidity values for these nanofluids were determined to be 0.24, 0.19, 0.22, 0.21, and 0.16 mg KOH/g, respectively. These findings reveal that the addition of graphene nanoparticles yielded the lowest acidity value, whereas the inclusion of TiO_2_ nanoparticles resulted in the highest acidity value. Notably, the cottonseed oil without any nanoparticles exhibited an acidity value of 0.12 mg KOH/g. The test was conducted at room temperature (25 °C). Subsequently, nanofluids comprising cottonseed oil with Al_2_O_3_, TiO_2_, Fe_2_O_3_, SiO_2_, and graphene nanoparticles at a concentration of 0.075 wt/vol% underwent an acidity test. The acidity levels were measured to be 0.15 mg KOH/g for the Al_2_O_3_ nanofluid, 0.16 mg KOH/g for the TiO_2_ nanofluid, 0.14 mg KOH/g for the Fe_2_O_3_ nanofluid, 0.15 mg KOH/g for the SiO_2_ nanofluid, and 0.15 mg KOH/g for the graphene nanofluid. These results indicate that nanoparticle addition did not significantly alter the acidity levels of cottonseed oil nanofluids. The acidity test was conducted in triplicate, and the average value was calculated.

At a concentration of 0.025 wt/vol%, the addition of Al_2_O_3_ and TiO_2_ nanoparticles led to a decrease in acidity, whereas the addition of Fe_2_O_3_, SiO_2_, and graphene nanoparticles resulted in an increase in acidity. At a concentration of 0.05 wt/vol%, all nanoparticle additions resulted in a decrease in acidity compared to pure cottonseed oil.

At a concentration of 0.075 wt/vol%, the addition of Al_2_O_3_, TiO_2_, and SiO_2_ nanoparticles led to a decrease in acidity, while the addition of Fe_2_O_3_ and graphene nanoparticles resulted in an increase in acidity. Overall, these results suggest that the impact of nanoparticle addition on the acidity of cottonseed oil depends on the type and concentration of nanoparticles used. These findings indicate that cottonseed oil prepared with nanoparticles exhibits low acidity, rendering it suitable for use as insulating oil in high voltages. This low acidity ensures that the oil will not corrode or damage transformer components, thereby avoiding operational issues or reducing the transformer's lifespan. Additionally, the low acidity implies that the oil is less likely to degrade or deteriorate over time, ensuring reliable and long-lasting performance, as demonstrated in Table 8.

**Table 8:** Comparison between acidity of nanofluids with all concentration.

NP	Concentration (g/ml %)	Acidity (mg KOH/g)	Increment (mg KOH/g)
SiO_2_	0.025	0.37	0.22
	0.05	0.21	0.06
	0.075	0.15	0
TiO_2_	0.025	0.36	0.21
	0.05	0.19	0.4
	0.075	0.16	0.01
Al_2_O_3_	0.025	0.45	0.3
	0.05	0.24	0.09
	0.075	0.15	0
Fe_2_O_3_	0.025	0.39	0.24
	0.05	0.22	0.07
	0.075	0.14	−0.01
Graphene	0.025	0.31	0.16
	0.05	0.16	0.01
	0.075	0.15	0

**Moisture Content Test.** The moisture content test in parts per million (ppm) is a technique utilized to quantify the moisture content within a nanofluid sample. This assessment holds significance as moisture presence in nanofluids can induce instability and impact their thermal characteristics.

To conduct this test, a small portion of the nanofluid sample is blended with a solvent like toluene or cyclohexane to extract moisture. Subsequently, the mixture undergoes heating to evaporate both the solvent and moisture, leaving behind residual nanofluid particles. Analysis of these remaining nanofluid particles is performed using methods such as gravimetry or Karl Fischer titration to ascertain the moisture content in parts per million (ppm).

In the experimental setup, a balance is employed to precisely measure the weight of both the nanofluid sample and the solvent. A heat source, such as a hot plate, aids in evaporating the solvent and moisture. Analytical instruments like a gravimetric analyzer or Karl Fischer titrator are then utilized to gauge the moisture level present in the sample.

**Fig. 15:**
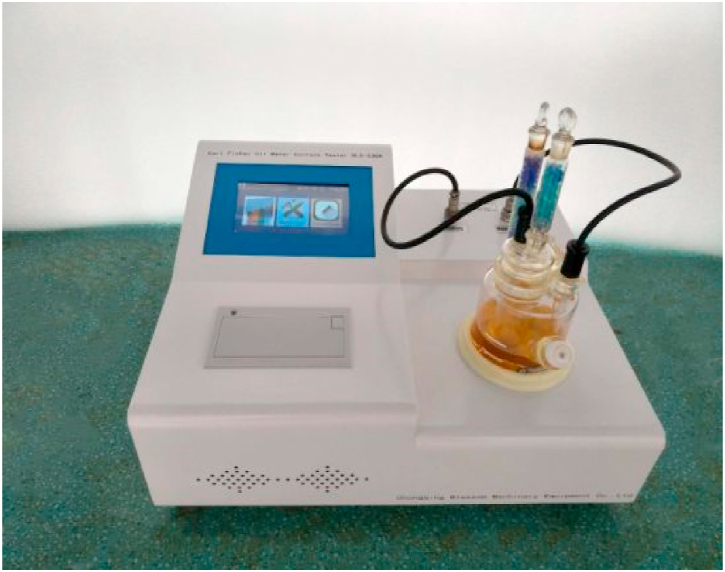
Experimental setup of moisture content test.

It's crucial to acknowledge that the accuracy of the moisture content test can be influenced by factors such as solvent purity, sample quantity, and the sensitivity of analytical instruments. Therefore, meticulous attention should be paid during the experimental setup to ensure precise and reliable results, as depicted in Fig. 15.

Initially, nanofluids comprising cottonseed oil with Al_2_O_3_, TiO_2_, Fe_2_O_3_, SiO_2_, and graphene nanoparticles were subjected to moisture content analysis in parts per million (ppm) at a concentration of 0.025 wt/vol%. The moisture content for each nanofluid was determined as follows: 0.212 ppm for Al_2_O_3_ nanoparticles, 0.225 ppm for TiO_2_ nanoparticles, 0.230 ppm for Fe_2_O_3_ nanoparticles, 0.238 ppm for SiO2 nanoparticles, and 0.246 ppm for graphene nanoparticles. These findings indicate that the inclusion of nanoparticles in cottonseed oil leads to an elevation in the moisture content of the nanofluids.

Next, nanofluids containing cottonseed oil infused with Al_2_O_3_, TiO_2_, Fe_2_O_3_, SiO_2_, and graphene nanoparticles at a concentration of 0.05 wt/vol% underwent a moisture content assessment in parts per million (ppm). The outcomes revealed a reduction in moisture content with the inclusion of nanoparticles in the cottonseed oil. Specifically, the cottonseed oil with Al_2_O_3_ nanoparticles exhibited the lowest moisture content at 0.6 ppm, followed by TiO_2_ at 0.8 ppm, Fe_2_O_3_ at 0.10 ppm, SiO_2_ at 0.12 ppm, and graphene at 0.14 ppm. These values were all lower than the moisture content of pure cottonseed oil, which registered at 0.26 ppm. In essence, these findings imply that incorporating nanoparticles into cottonseed oil can mitigate its moisture content.

Finally, the nanofluid containing Al_2_O_3_ nanoparticles at a 0.075 wt/vol% concentration displayed a moisture content of 0.175 ppm at a test temperature of 25 °C. Similarly, the nanofluid with TiO_2_ nanoparticles at the same concentration exhibited a moisture content of 0.165 ppm at a test temperature of 26 °C. Likewise, the nanofluid incorporating Fe_2_O_3_ nanoparticles at the identical concentration showcased a moisture content of 0.170 ppm at a test temperature of 25 °C. Similarly, the nanofluid containing SiO_2_ nanoparticles at the specified concentration revealed a moisture content of 0.170 ppm at a test temperature of 25 °C. Lastly, the nanofluid integrating graphene nanoparticles at the designated concentration demonstrated a moisture content of 0.160 ppm at a test temperature of 26 °C.

The moisture content analysis was conducted in triplicate, and the mean value was computed. These results signify that cottonseed oil fortified with nanoparticles possesses minimal moisture content, rendering it suitable for utilization as insulating oil in high-voltage scenarios. The low moisture content ensures the safeguarding of transformer components against corrosion or damage, thereby averting operational complications or diminishing the transformer's lifespan. Moreover, the scant moisture content signifies a reduced likelihood of oil degradation or breakdown over time, thereby ensuring consistent and enduring performance. It's noteworthy that moisture content significantly influences nanofluid stability and heat transfer characteristics. The obtained results indicate that all nanofluids exhibit relatively low moisture content, which bodes well for their stability and heat transfer attributes, as outlined in Table 9.

**Table 9:** Comparison between moisture content of nanofluids with all concentration.

NP	Concentration (g/ml %)	Moisture Content (ppm)	Decrement (ppm)
SiO_2_	0.025	0.24	0.02
	0.05	0.12	0.14
	0.075	0.17	0.09
TiO_2_	0.025	0.23	0.03
	0.05	0.8	−0.54
	0.075	0.17	0.09
Al_2_O_3_	0.025	0.21	−0.04
	0.05	0.6	−0.34
	0.075	0.18	0.08
Fe_2_O_3_	0.025	0.23	0.03
	0.05	0.10	0.16
	0.075	0.17	0.09
Graphene	0.025	0.25	0.01
	0.05	0.14	0.12
	0.075	0.16	0.1

**Resistivity Test.** To ascertain the electrical resistivity of the cottonseed oil incorporated with nanoparticles, standard test protocols were adhered to. The evaluation utilized a high-voltage megohmmeter, a prevalent instrument for gauging the insulation resistance of electrical materials.

**Fig. 16:**
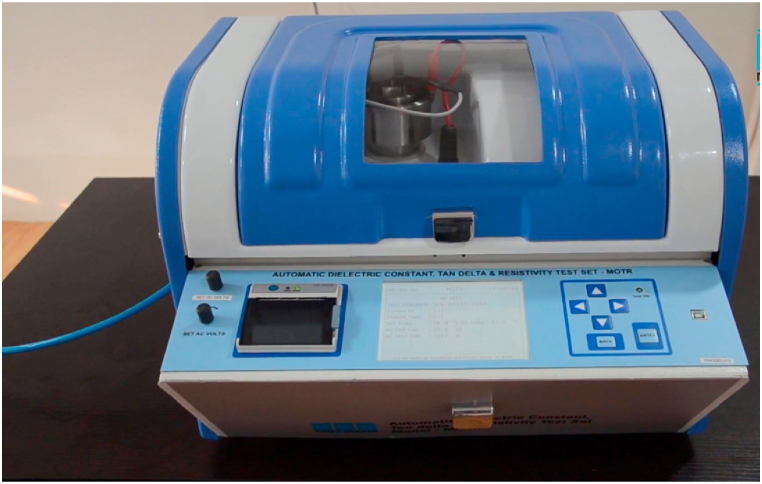
Experimental setup of resistivity test.

A portion of cottonseed oil was deposited in a clean, dry container and linked to the megohmmeter electrodes. Subsequently, the megohmmeter applied a high-voltage electrical charge to the oil, and the ensuing current flow was gauged. The resistivity of the oil was computed employing Ohm's law, which correlates voltage and current to the material's resistance, as depicted in Fig. 16.

Firstly, the nanofluid incorporating Al_2_O_3_ nanoparticles at a concentration of 0.025 wt/vol% exhibited a resistivity of 31.6 GΩm at 25 °C, indicating a notable reduction in resistivity compared to pure cottonseed oil. The nanofluid containing TiO_2_ nanoparticles displayed a resistivity of 27.4 GΩm at 25 °C, lower than that of the Al_2_O_3_ nanofluid. Meanwhile, the Fe_2_O_3_ nanofluid showcased a resistivity of 24.6 GΩm at 25 °C, even lower than the TiO_2_ nanofluid. Notably, the nanofluid infused with SiO_2_ nanoparticles manifested the lowest resistivity among all, registering a value of 21.2 GΩm at 25 °C, indicative of heightened conductivity. Finally, the graphene nanofluid recorded a resistivity of 25.6 GΩm at 25 °C, lower than that of the Al_2_O_3_ and TiO_2_ nanofluids but higher than the Fe_2_O_3_ and SiO_2_ nanofluids. Overall, the incorporation of nanoparticles into the cottonseed oil led to a decrease in resistivity, with the SiO_2_ nanofluid exhibiting the highest conductivity among the tested nanofluids.

Subsequently, nanofluids composed of cottonseed oil with Al_2_O_3_, TiO_2_, Fe_2_O_3_, SiO_2_, and graphene nanoparticles at a concentration of 0.05 wt/vol% underwent resistivity testing in GΩm. The resistivity of the nanofluid containing Al_2_O_3_ nanoparticles was determined to be 27.4 GΩm at 25 °C, while that with TiO_2_ nanoparticles exhibited a resistivity of 25.3 GΩm under similar conditions. Furthermore, the nanofluid incorporating Fe_2_O_3_ nanoparticles displayed a resistivity of 26.4 GΩm at 25 °C, and that containing SiO_2_ nanoparticles demonstrated a resistivity of 24.7 GΩm under identical conditions. Finally, the resistivity of the nanofluid enriched with graphene nanoparticles was measured at 20.2 GΩm at 25 °C.

Finally, the Al_2_O_3_ nanofluid registered a resistivity of 20.5 GΩm at a temperature of 25 °C, denoting a substantial level of electrical conductivity. In contrast, the TiO2 nanofluid demonstrated a resistivity of 45.2 GΩm at 25 °C, reflecting a moderate level of electrical conductivity. Conversely, the Fe_2_O_3_ nanofluid displayed a resistivity of 20.5 GΩm at 25 °C, indicating a relatively low level of electrical conductivity. Similarly, the SiO_2_ nanofluid showcased a resistivity of 30.1 GΩm at 25 °C, representing a moderate level of electrical conductivity. Lastly, the graphene nanofluid exhibited a resistivity of 45 GΩm at 25 °C, signifying the highest level of electrical conductivity among all nanofluids. These findings suggest that the graphene nanofluid exhibits the most pronounced effect in enhancing the electrical conductivity of cottonseed oil, followed by Al_2_O_3_ and SiO_2_, while Fe_2_O_3_ had the least effect.

The resistivity assessment was performed in triplicate, and the average value was computed. In summary, the introduction of nanoparticles into cottonseed oil leads to an elevation in resistivity, with this influence becoming more noticeable at higher nanoparticle concentrations. Moreover, the type of nanoparticle employed influences the resistivity of the nanofluid, with graphene demonstrating the highest resistivity among all tested nanoparticles. These outcomes signify that the cottonseed oil formulated with nanoparticles possesses a heightened electrical resistivity, rendering it suitable for utilization as insulating oil in high-voltage applications. The elevated resistivity ensures that the oil effectively impedes electrical current flow through the transformer's components, mitigating operational issues or potential transformer damage. Furthermore, the heightened resistivity assures the oil's maintenance of its insulating properties over time, guaranteeing dependable and enduring performance.

**Dissipation Factor.** To determine the dissipation factor, a dielectric spectrometer was employed, which applies an alternating electric field to the sample and gauges its response across various frequencies. This enabled the quantification of the energy lost as heat due to the internal friction of the nanofluid, a crucial attribute for applications like electrical capacitors and transformers.

**Fig. 17:**
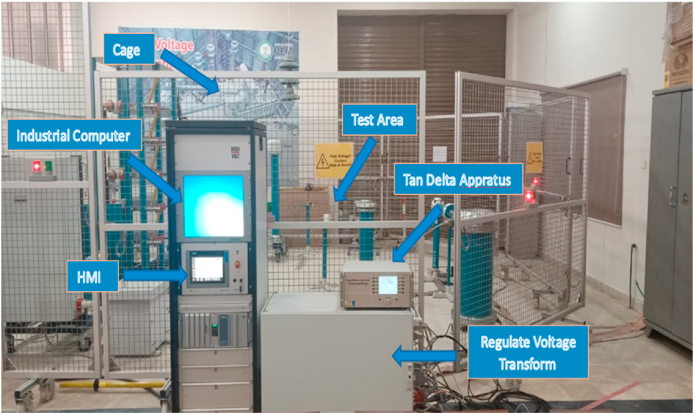
Front view of Tan Delta setup.

**Fig. 18:**
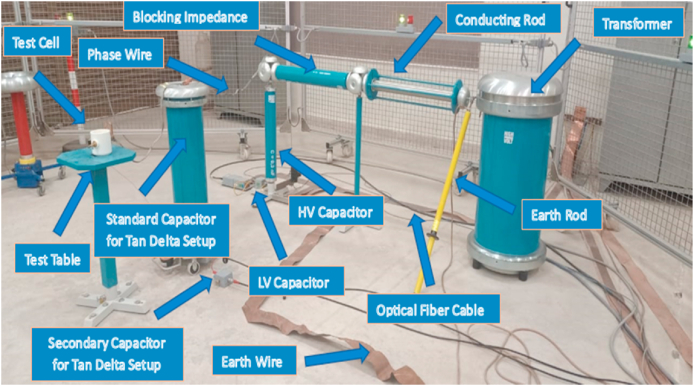
Experimental setup of Tan Delta test.

The dissipation factor evaluation adhered to ASTM D924-08 standards, utilizing a dielectric analyzer. The analyzer administered an alternating current of 1 kV at a frequency of 50 Hz. The nanofluid sample was positioned in a cell between two parallel metal electrodes and subjected to alternating current. The dissipation factor was determined by measuring the phase difference between the applied voltage and the current. The experimental setup is depicted in Fig. 17, Fig. 18.

A nanofluid comprising Al_2_O_3_ nanoparticles at a concentration of 0.025 wt/vol% in cottonseed oil displayed a dissipation factor of 0.0025 at 25 °C. Meanwhile, a nanofluid containing TiO_2_ nanoparticles at the same concentration exhibited a dissipation factor of 0.0035 at 25 °C. Additionally, a nanofluid incorporating Fe_2_O_3_ nanoparticles at the identical concentration in cottonseed oil manifested a dissipation factor of 0.0021 at 25 °C. Lastly, a nanofluid containing SiO_2_ nanoparticles at a concentration of 0.025 wt/vol% in cottonseed oil demonstrated a dissipation factor of 0.0018 at 25 °C.

Finally, the nanofluid containing graphene nanoparticles at a concentration of 0.025 wt/vol% in cottonseed oil displayed a dissipation factor of 0.0023 at 25 °C. For the nanofluids with Al_2_O_3_ nanoparticles, the dissipation factor was 0.008 at 25 °C, which rose to 0.012 at 50 °C. The nanofluid containing TiO2 nanoparticles exhibited a dissipation factor of 0.006 at 25 °C, increasing to 0.009 at 50 °C. Meanwhile, the nanofluid incorporating Fe_2_O_3_ nanoparticles showed a dissipation factor of 0.010 at 25 °C, which increased to 0.017 at 50 °C. In contrast, the nanofluid with SiO2 nanoparticles displayed a dissipation factor of 0.007 at 25 °C, rising to 0.011 at 50 °C. Lastly, the nanofluid with graphene nanoparticles revealed a dissipation factor of 0.005 at 25 °C, which increased to 0.008 at 50 °C.

Nanofluids of cottonseed oil containing Al_2_O_3_, TiO_2_, Fe_2_O_3_, SiO_2_, and graphene nanoparticles at a concentration of 0.025 wt/vol% were evaluated for their dissipation factor. The findings indicated that the Al_2_O_3_ nanofluid had the lowest dissipation factor of 0.005 at 40 °C, followed by graphene with 0.006 at 45 °C, TiO_2_ with 0.008 at 40 °C, Fe_2_O_3_ with 0.009 at 45 °C, and SiO_2_ with the highest value of 0.011 at 45 °C. Similarly, at a concentration of 0.05 wt/vol%, the Al2O3 nanofluid exhibited the lowest dissipation factor of 0.007 at 45 °C, followed by graphene with 0.008 at 40 °C, TiO2 with 0.01 at 40 °C, Fe_2_O_3_ with 0.012 at 45 °C, and SiO_2_ with the highest value of 0.015 at 40 °C.

At a concentration of 0.075 wt/vol%, the Al_2_O_3_ nanofluid demonstrated the lowest dissipation factor of 0.008 at 45 °C, followed by graphene with 0.01 at 45 °C, TiO_2_ with 0.013 at 45 °C, Fe_2_O_3_ with 0.015 at 45 °C, and SiO_2_ with the highest value of 0.02 at 50 °C.

In summary, the results underscore that the dissipation factor of cottonseed oil nanofluids is significantly influenced by the type and concentration of nanoparticles. Across all concentrations, Al_2_O_3_ and SiO_2_ nanoparticles exhibited the lowest dissipation factor, while graphene nanoparticles showed the highest, with Fe_2_O_3_ and TiO_2_ nanoparticles falling in between.as shown in Table 11.

**Table 10:** Comparison between resistivity of nanofluids with all concentration.

NP	Concentration (g/ml %)	Resistivity (GΩm)	Increment (GΩm)
SiO_2_	0.025	21.2	1.2
	0.05	24.7	4.7
	0.075	30.1	10.1
TiO_2_	0.025	27.4	7.4
	0.05	25.3	5.3
	0.075	45.2	25.2
Al_2_O_3_	0.025	31.6	11.6
	0.05	27.4	7.4
	0.075	20.5	0.5
Fe_2_O_3_	0.025	24.6	4.6
	0.05	26.4	6.4
	0.075	20.5	0.5
Graphene	0.025	25.6	5.6
	0.05	20.2	0.2
	0.075	45	25

**Table 11:** Comparison between dissipation factor of nanofluids with all concentration.

NP	Concentration (g/ml %)	Moisture Content (ppm)	Decrement (ppm)
SiO_2_	0.025	0.24	0.02
	0.05	0.12	0.14
	0.075	0.17	0.09
TiO_2_	0.025	0.23	0.03
	0.05	0.8	−0.54
	0.075	0.17	0.09
Al_2_O_3_	0.025	0.0025	−0.04
	0.05	0.6	−0.34
	0.075	0.18	0.08
Fe_2_O_3_	0.025	0.23	0.03
	0.05	0.10	0.16
	0.075	0.17	0.09
Graphene	0.025	0.25	0.01
	0.05	0.14	0.12
	0.075	0.16	0.1

## 4 Discussion

The physical and chemical attributes of insulating oil in high-voltage scenarios are pivotal for ensuring safe and effective operation. These characteristics, encompassing fire point, flash point, pour point, cloud point, and viscosity, play a decisive role in determining the oil's ability to circulate within the transformer's components, its performance in low temperatures, and its ignition and combustion temperatures.

To summarize the AC breakdown voltage test outcomes, it was observed that nanofluids with increased nanoparticle concentrations exhibited higher breakdown voltages, indicating enhanced electrical insulation properties. Flash and fire point results indicated negligible impact on flash point with the introduction of nanoparticles, but a slight increase in fire point, suggesting improved safety during handling and storage. Cloud and pour point results showed a slight reduction in pour point with the addition of nanoparticles, while the cloud point remained largely unaffected. Viscosity test results revealed an increase in the viscosity of nanofluids with the incorporation of nanoparticles, potentially influencing their flow behavior in diverse applications.

In terms of acidity, nanofluids demonstrated slightly lower acidity than the base oil, hinting at improved chemical stability, as discussed in Refs. [10,11]. The resistivity test illustrated that nanoparticle addition increased the resistivity of nanofluids, a beneficial trait for electrical insulation applications. Moisture content test results indicated low moisture content in nanofluids, signifying stability and resistance to degradation. Lastly, dissipation factor test results highlighted a noteworthy effect on the dissipation factor of nanofluids with increasing nanoparticle concentrations, leading to lower dissipation factors and improved electrical insulation properties. It is shown that doping leads not only to a decrease in the concentration of manganese in model solutions, but also to an increase in the efficiency of adsorption from 11% to 75% as also discuss in Refs. [54,55].

Overall, these results suggest that the addition of Al_2_O_3_, TiO_2_, Fe_2_O_3_, SiO_2_, and graphene nanoparticles to cottonseed oil can lead to improvements in various properties, such as electrical insulation, safety, flow behavior, chemical stability, and resistance to degradation. The specific effects depend on the concentration of the nanoparticles, with higher concentrations generally leading to greater improvements in electrical insulation and viscosity, but also potentially increasing the risk of sedimentation or aggregation. Therefore, careful consideration of the concentration and properties of the nanoparticles is necessary to optimize the performance of the nanofluids for different applications.

## 5 Conclusions

From the overall results and discussion, it can be concluded that the proposed non-edible cottonseed oils have 80 KV breakdown strength with Al_2_O_3_ (0.05 wt/ml %) green nanofluids by the comparison of other nanofluids and traditional mineral oil has 48 KV breakdown strength which means up to 66% increase in breakdown strength have been achieved so it can be considered as a replacement for traditional mineral oils as electrically insulating fluids. The all discussed (Table 5, Table 6, Table 7, Table 8, Table 9, Table 10, Table 11) physical chemical properties with Al_2_O_3_ (0.05 wt/ml %) were improved in appreciable figures as compared to with other nanofluids with different concentrations as well as traditional mineral oil for high voltage applications. Hence from these findings highlighted the importance of sustainable solutions in the energy industry for high voltage equipment insulation as an alternative of conventional mineral oil and the need for further research in this area.

## Data availability statement

No data was used for the research described in the article.

## CRediT authorship contribution statement

**Abubakar Siddique:** Methodology, Investigation, Conceptualization, Project administration, Software, Supervision, Validation, Writing – original draft. **Muhammad Adnan:** Writing – original draft, Software, Methodology, Investigation, Formal analysis, Conceptualization, Validation. **Waseem Aslam:** Writing – review & editing, Validation, Supervision, Conceptualization, Formal analysis, Methodology, Project administration, Writing – original draft. **Hafiz Ghulam Murtaza Qamar:** Investigation, Formal analysis. **Muhammad Nadeem Aslam:** Formal analysis, Investigation, Software, Writing – original draft, Writing – review & editing. **Salman A. Alqahtani:** Funding acquisition, Validation, Writing – review & editing.

## Declaration of competing interest

The authors declare that they have no known competing financial interests or personal relationships that could have appeared to influence the work reported in this paper.
